# Service selection in mobile environments: considering multiple users and context-awareness

**DOI:** 10.1080/12460125.2018.1513223

**Published:** 2018-09-12

**Authors:** Bernd Heinrich, Michael Mayer

**Affiliations:** Institute of Management Information Systems, University of Regensburg, Regensburg, Germany

**Keywords:** Service selection, multi-user processes, context information, mobile environment

## Abstract

In mobile environments, users often need to coordinate their actions with other users with regard to user-individual context information like current location when selecting suitable services for a process. Thereby, some users may prefer to conduct particular services together with certain other users. Such multi-user context-aware service selections could result in complex decision problems – making decision support for the participating users highly valuable or even necessary. To do so, we propose an optimisation-based service selection approach for multi-user context-aware processes. We also show how our approach provides decision support by evaluating its efficacy based on a real-world scenario.

## 1. Introduction

The tremendous advances in mobile technologies and the rise of mobile business over the last decade have led to a rapid growth of the service market (Statista, 2017). Selecting services for processes in mobile environments like a tourism city day trip often results in a decision problem of high complexity as it is often necessary to coordinate the actions of multiple users as well as to consider context information. In this regard, context information can refer to the current location, daytime, and so on, or generally speaking ‘any information that can be used to characterize the situation of an entity^1^’ (Dey, 2001, p. 5). Such multi-user context-aware processes in mobile environments can be found, for example, in roadside, healthcare or disaster relief assistance, the areas of everyday efficiency and planning (price comparison, routing, schedule management on mobile devices), or in the tourism domain (cf. Gavalas, Konstantopoulos, Mastakas, & Pantziou, 2014; Neville et al., 2016; Ventola, 2014; Zhang, Adipat, & Mowafi, 2009).

Considering, for instance, healthcare assistance in hospitals, healthcare professionals need to be assigned to patients in a suitable way to adequately support their therapy, where some patients need multiple treatments (i.e. services) in a defined order (i.e. process) (cf. Marynissen & Demeulemeester, 2016). Here, healthcare professionals currently start to use mobile devices in combination with hospital information systems to retrieve information about patients such as medical data and previous diseases but also about treatment rooms and operating theatres in terms of context information like location and time schedule (cf. Boruff & Storie, 2014; Ventola, 2014). This information can then be used for assigning healthcare professionals with certain skills to patients and near-located, available treatment rooms/operating theatres to minimise the overall duration (including waiting time) for the patients, for instance. Consequently, healthcare professionals need to conduct certain actions to treat patients in the best way. For some of these actions, it is more beneficial when they are conducted together by several healthcare professionals with different skills (e.g. surgery) – requiring the coordination of the healthcare professionals. This can be characterised as a multi-user context-aware service selection problem focusing on the support of patients’ medical therapy where the respective selection (i.e. assignment) is highly complex (cf. Marynissen & Demeulemeester, 2016).

Another application field for multi-user context-aware processes in a mobile environment is the tourism domain, for instance, a city day trip conducted together by a group of users. Here, the users can retrieve information about real-world entities like sights, restaurants or museums by using mobile information applications (e.g. Yelp, TripAdvisor) – where each entity with its properties (e.g. price, duration, location, business hours) can be understood as a service object (cf. Lewerenz, 2015; Yu & Reiff-Marganiec, 2009). Such a city day trip usually encompasses many different actions like visiting a museum, having lunch and visiting a sight. Each of these actions could then be realised by different real-world entities represented by service objects, for example, ‘Pinakothek of Modern Art’ or ‘Bavarian National Museum’ (referring to the city of Munich, Germany). Selecting suitable service objects for such a process (i.e. trip) requires to deal with the preferences (e.g. price more important than duration) and requirements (e.g. overall budget) of each individual user as well as with the context information of both the users (location, daytime, etc.) and the real-world entities (location, business hours, etc.). Moreover, with several users conducting a city day trip together finding the optimal composition of service objects for each user additionally requires a coordination of the users’ actions in their processes. Thereby, when dealing with multiple users in service selection, we need to cope with (inter-)user preferences, which we denote as Inter-User-Requests (IUR). An example for an IUR here would be a user favouring to visit the ‘Bavarian National Museum’ together with two other particular users participating in the trip. Thus, in addition to context information, we also consider such IUR in this work that means user-defined requests referring to other users.

Against this background, users trying to determine their optimal composition of services resp. service objects to conduct a multi-user context-aware process are usually confronted with an information overload problem (cf. Shen, Wang, Tang, Luo, & Guo, 2012; Zhang et al., 2009) since there often exist many alternative service objects for realising each action of such a process (referring to the example above, TripAdvisor lists over 3,000 different restaurants for having lunch in Munich^2^). More precisely, when taking into account multiple users and context information, a service selection problem of high complexity results since it requires to consider dependencies that exist within a user’s service composition as well as among different users’ service compositions. These dependencies are illustrated in more detail in the next section. As a consequence, a suitable approach is needed to support the users in terms of selecting the optimal service composition for each user. To the best of our knowledge, none of the existing optimisation-based service selection approaches aims at integrating multiple users and context-awareness (cf. section 3.1 Related Literature). This leads us to the following research question for our paper:

How to develop an optimisation-based service selection approach which considers dependencies resulting from both multiple users and context information?

In the following section, we present a motivating scenario for our research which is followed by the background in terms of a discussion of related literature, the resulting research gap, our contribution, and the introduction of our model setup. In the fourth section, we analyse and model both multiple users and context information. Based on that, we propose our approach in terms of an optimisation model (cf. Section 5), which we then evaluate regarding efficacy and performance. Finally, we conclude our paper with a discussion on implications (Section 7), important limitations and an outlook on further research (Section 8).

## 2. Motivating scenario

**Figure 1.**
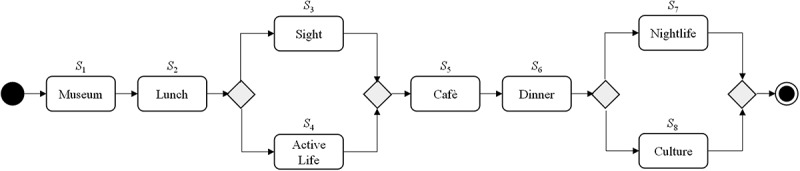
Process model for city day trip.

Our scenario refers to a tourism day trip to the City of Munich, Germany, by three individual users where the users plan to conduct several different actions such as visiting a museum, having lunch or visiting a café (cf. Figure 1 for an example). Obviously, there exist numerous alternatives for conducting each action (e.g. Restaurant ‘Vinaiolo’, Restaurant ‘L’Ancora’, etc.). Subject to the individual price, duration and location of these alternatives, some of them are more valuable for a user than others based on her/his own individual target weights (e.g. price may be more important than duration) and requirements (e.g. overall budget). Furthermore, in such a scenario, it is likely that some users also have requests that refer to other users (i.e. IUR), for example, ‘user 3 requests to take a coffee together with user 2 regardless which café’ or ‘user 1 requests not to go all together to the “German Theatre Munich”’. Taking the first IUR, user 3 associates a positive value for being at the same café at the same time as user 2. Moreover, as some museums or sights offer group discounts, it could be more beneficial for the three users to visit the same museum or sight.

Obviously, due to the high number of available real-world entities, individual target weights and requirements as well as IUR, decision support is valuable to determine the best entities for the complete day trip regarding all users. Therefore, we represent each entity (e.g. Restaurant ‘Vinaiolo’) and its information such as price, duration, business hours and location as service object with non-functional properties (NFP). Based on this, service selection can be used to determine the optimal set of service objects (i.e. service composition) for each user and the entire process.

Regarding the above-mentioned IUR ‘user 3 requests to take a coffee together with user 2 regardless which café’, the realisation of the positive value associated by user 3 supposes that the selected service compositions of both users encompass the same café (represented by the same service object). However, if the service objects are selected independently for both users, this would obviously only happen by chance. Therefore, realising IUR requires to take such *preference-based dependencies* between different users’ service compositions into account when selecting suitable service objects (cf. Heinrich, Klier, Lewerenz, & Mayer, 2015). Additionally, both users must arrive at the café at the same time, which is dependent on the individual starting time of the day trip of both users (i.e. initial context of each user) and the duration of the previously conducted actions which is most likely different for each of them. In this respect, it may also be beneficial for one user to wait a certain amount of time to be able to visit the same café at the same time as the other user and thus realising the IUR. To consider such *temporal-based dependencies*, a temporal coordination of the users’ actions including possible waiting times is necessary, too. The same applies to *context-based dependencies* that result from context information such as group discounts or the distance to cover between, for example, the café visited by both users and the preceding actions each user has conducted.

To sum up, in order to provide feasible and suitable decision support in such a multi-user context-aware scenario, all these different types of *preference-based, context-based* and *temporal-based dependencies* must be taken into account when selecting the optimal service compositions for all users.

## 3. Background

Next, we review existing works and based on that discuss both our research gap and contribution. This will be followed by the introduction of our model setup.

### 3.1. Related literature

We structure the existing literature dealing with multiple users and context-awareness in optimisation-based service selection according to the types of *preference-based, context-based* and *temporal-based dependencies* introduced above.

First, we analyse *preference-based dependencies* resulting from multiple users. In this respect, existing multi-user service selection approaches deal with restrictions that prescribe or limit the usage of services (or service objects) by two or more users. Those so-called hard restrictions must be satisfied in a feasible service composition. For example, Benouaret, Benslimane, and Hadjali (2012), Wanchun, Chao, Xuyun, and Chen (2011) and Wang et al. (2010) examine a situation, where the mutual usage of a certain service by several users is mandatory, while He, Han, Yang, Grundy, and Jin (2012), Kang, Liu, Tang, Liu, and Fletcher (2011) and Wang, Hsu, Liang, Sun, and Yang (2014) address capacity limits of services. However, preference-based dependencies and thus users preferring (but not enforcing) to use certain services (or service objects) together with other users have not been addressed in literature so far.

Second, when considering *context-based dependencies*, there are many works that deal with context information and context-awareness in terms of selecting (single) services for a single user (e.g. Ai & Tang, 2008; Deng, Huang, Hu, Zhao, & Wu, 2016; Sandionigi, Ardagna, Cugola, & Ghezzi, 2013; Vanrompay, Pinheiro, & Berbers, 2009; Yu & Reiff-Marganiec, 2009; Zhou, Zheng, Song, Du, & Chen, 2008). Few of them also consider context-based dependencies that could exist within a certain part or the entire service composition of a user (e.g. Deng et al., 2016; Shen et al., 2012; Xu & Jennings, 2010; Yu & Reiff-Marganiec, 2009; Zhou et al., 2008). However, those approaches focus solely on a single user and thus on context-based dependencies within a single user’s service composition. But as we consider multi-user processes, we must account for the fact that there could also exist context information referring to multiple users.

Third, when addressing both (time-dependent) preferences/IUR and (time-dependent) context information, we additionally need to deal with *temporal-based dependencies*. Optimisation-based service selection approaches coping with such temporal-based dependencies can be found in (Guidara, Guermouche, Chaari, Tazi, & Jmaiel, 2014; Heinrich & Lewerenz, 2015; Xu & Jennings, 2010). Although they define a time concept, none of them addresses a temporal coordination of the users’ actions including possible waiting times. In this regard, the consideration of waiting times is necessary for comprehensive decision support as, for instance, this allows one or many users to wait instead of moving to a less favoured service (or service object).

### 3.2. Identified research gap and contribution

In summary, important contributions have been made with respect to multiple users and context-awareness in service selection. However, an optimisation-based service selection approach that copes with *preference-based, context-based* and *temporal-based dependencies* is – to the best of our knowledge – missing so far. Thus, we will address this gap in our work in terms of proposing a novel service selection approach.

Existing optimisation-based approaches, which solve the general service selection problem (i.e. without considering multiple users and context information), search for the optimal service composition for one single user under consideration of target weights and requirements regarding the NFP like price, availability, and so on (e.g. Alrifai, Risse, & Nejdl, 2012; Ardagna & Pernici, 2007; Yu, Zhang, & Lin, 2007; Zeng et al., 2004). At this, the service selection problem is usually formulated as knapsack optimisation problem (e.g. Alrifai & Risse, 2009; Alrifai et al., 2012; Yu et al., 2007). However, when considering multiple users and context information, we have to deal with the question how to model and integrate the resulting preference-based, context-based and temporal-based dependencies in terms of an optimisation-based approach. Here the literature provides two fundamental alternatives: a stateless versus stateful representation of dependencies. In terms of a stateless representation, dependencies are integrated directly into an optimisation model. For instance, He et al. (2012), Jin, Zou, Yang, Lin, and Shuai (2012) and Kang et al. (2011) consider multiple users and capacity limits by extending the optimisation model in terms of additional constraints. However, they only focus on hard restrictions. Regarding a stateful representation, first approaches (e.g. Lewerenz, 2015) utilise the concept of world and belief states (cf. Ghallab, Nau, & Traverso, 2004) to organise and model context information. Thus, existing context-based dependencies are specified by state-service combinations that are determined before the optimisation takes place. However, they do not consider preference-based and temporal-based dependencies in their approaches.

We aim to provide both a stateless and stateful optimisation model, each incorporating dependencies resulting from multiple users and context information. This allows us to evaluate both alternatives and their advantages resp. disadvantages in detail. In conclusion, this leads us to the following three-fold contribution of our paper:

① Consideration of *preference-based* and *context-based dependencies* resulting from multiple users and context information

② Consideration of *temporal-based dependencies* resulting from time-dependent preferences/IUR and time-dependent context information which requires a time concept dealing especially with waiting times

③ Development of *optimisation models* for a multi-user context-aware service selection based on a stateful resp. stateless representation of dependencies

### 3.3. Model setup

In this section, we introduce our model setup, referring to those definitions and modelling elements in line with existing works that can serve as a common knowledge base. This allows for a better differentiation between existing knowledge and our contribution ①–③ in the Sections 4 and 5.

We consider a sequential process that consists of a number of actions or service classes $S_{i}$ (with i = 1 to I). Each service class encompasses a set of functional equivalent services $s_{ij}$ (with j = 1 to $J_{i}$) – which are referred to as service objects – that differ only in their NFP. Furthermore, a service composition is defined as a concrete implementation of a process in terms of a set of service objects with exactly one service object out of each service class of the process. Appendix A provides an overview of the used formal notation throughout this work.

When considering service selection without dealing with context information, a service object $s_{ij}$ would be described only by the set M of non-context-aware (NCA) attributes like price or duration. Based on that, the vector $q_{ij}={\left[q_{ij}^{1},\ldots ,q_{ij}^{M}\right]}^{T}$ contains the quantified NFP values of a service object $s_{ij}$ regarding all NCA attributes M. For the selection of service objects with several NFP, a utility function U is often used – where the purpose of U is to map the values of the different attributes onto a single utility value. In our work, we apply – in line with, for instance, Alrifai et al. (2012), Jin et al. (2012) and Guidara et al. (2014) – the utility function described in detail by Alrifai and Risse (2009). But without limitations, other utility functions could be used as well with our approach as the exact way the utility of a certain service object is calculated has no impact on the formulation of our optimisation models in Section 5. To determine the utility value of a service object, this utility function uses the simple additive weighting (SAW) technique consisting of normalisation and weighting of the NFP. For the normalisation step (i.e. to enable comparability between different NFP), the utility function utilises the aggregated minimum and maximum values of the attributes over all service classes $S_{i}$. Further, the attributes ∝∈M can be divided into the subset of attributes $M^{-}$ that need to be minimised and the subset of attributes $M^{+}$ that need to be maximised. The aggregated values $P_{min}^{\propto }$ and $P_{max}^{\propto }$ for each attribute ∝ in $M^{-}$ and $M^{+}$ can be calculated as follows:

$$\;\;\;\;\;\;\;\;\;\;\;\;\;\;\;\;\;\;\;\;\;\;\;\;\;\;\;\;\;\;\;\;\;\;\;\;\;\;\;\;\;\;\;\;\;\;\;\;\;\;\;\;\;P_{min}^{\propto }=\;\overset{I}{\underset{i=1}{\sum }}\left(P_{i,\;min}^{\propto }\right)with\;P_{i,\;min}^{\propto }=\underset{s_{ij}\in S_{i}}{\min}q_{ij}^{\propto }\;\;\;\;\;\;\;\;\;\;\;\;\;\;\;\;\;\;\;\;\;\;\;\;\;\;\;\;\;\;\;\;\;\;\;\;\;\;\;\;\;\;\;\;\;\;\;\;\;\;(1{)}^{3}$$

$$P_{max}^{\propto }=\;\overset{I}{\underset{i=1}{\sum }}\left(P_{i,\;max}^{\propto }\right)with\;P_{i,\;max}^{\propto }=\underset{s_{ij}\in S_{i}}{\max}q_{ij}^{\propto }$$

These aggregated minima and maxima could then be used to normalise the NFP values. To achieve a single utility value $U_{ij}$ (cf. Equation (2)) for a service object $s_{ij}$, the weighted sum over all attributes based on user-defined target weights $w^{\propto }$ regarding the attributes ∝∈M is determined. Here, it must hold that $\overset{M}{\underset{\propto =1}{\sum }}w^{\propto }=1$. Considering multi-user service selection and therefore multiple users a∈A leads to possibly varying utility values $U_{a_{ij}}$ of a particular service object $s_{ij}$ for different users a since each user is likely to have its own target weights $w_{a}^{\propto }$ (cf. Alrifai et al., 2012; Jin et al., 2012):

(2) $$U_{a_{ij}}=\underset{\propto \in M^{-}}{\sum }\left(\frac{P_{i,\;max}^{\propto }-q_{ij}^{\propto }}{P_{max}^{\propto }-P_{min}^{\propto }}\right)*w_{a}^{\propto }+\underset{\propto \in M^{+}}{\sum }\left(\frac{q_{ij}^{\propto }-P_{i,\;min}^{\propto }}{P_{max}^{\propto }-P_{min}^{\propto }}\right)*w_{a}^{\propto }$$

Based on this, the overall utility value of a service composition can be calculated by summing up the individual utilities of all selected service objects. Besides the target weights $w_{a}^{\propto }$, user-defined requirements in terms of global end-to-end constraints $Q_{a}^{\propto }$ regarding the NFP must be considered as well (e.g. Jin et al., 2012; Yu et al., 2007).

Now, when additionally considering context information in service selection, we distinguish whether this context information is of static or dynamic nature. In contrast to the static nature (i.e. the context information is exogenously given regarding the service composition, like weather), we speak of the dynamic nature of context information when the set of selected service objects influences the actual manifestation of the context information (cf. Damián-Reyes, Favela, & Contreras-Castillo, 2011; Vanrompay et al., 2009). Examples for such context information are daytime-dependent availability of service objects (i.e. business hours), price discount on a certain set of service objects, and the distance between different service providers or devices (Shen et al., 2012; Yu & Reiff-Marganiec, 2009; Zheng, Zhang, & Lyu, 2014; Zhou et al., 2008). Addressing this dynamic nature of context information leads to context-based dependencies between several or all service objects (Heinrich & Lewerenz, 2015; Zhou et al., 2008).

In service selection, context information can be taken into account by means of context-aware (CA) attributes (cf. Ai & Tang, 2008; Xu & Jennings, 2010; Yu & Reiff-Marganiec, 2009; Zhou et al., 2008) that together with the NCA attributes describe the NFP of a service object. Moreover, the subset of NCA attributes M and the subset of CA attributes O form together the set of attributes N (with M∪O=N and M∩O=∅) that are considered in a certain service selection problem. Furthermore, each user has her/his individual target weights $w_{a}^{\propto }$ and global end-to-end constraints $Q_{a}^{\propto }$ regarding all CA attributes O. But in contrast to NCA attributes, CA attributes are subject to the following three fundamental effects as a result of the existing *context-based dependencies* between different service objects (cf. Lewerenz, 2015):

1. The determination of context information is dependent on the service objects selected for a specific service composition. Thus, the *quantified values* of a CA attribute could be different for the same considered service object used in different service compositions.
2. As a direct consequence of (1), the utility of a service object or a set of service objects is affected by context information, which means the corresponding *utility value* is different for each service composition (thus influencing the selection of the *optimal composition*).
3. Furthermore, the selection of a service object could also have an effect on the *feasibility* of other service objects.

Consequently, all three fundamental effects need to be taken into account when modelling dependencies in the following.

## 4. Modelling preference-based, context-based and temporal-based dependencies

Based on the model setup, we will analyse and model *preference-based, context-based* and *temporal-based dependencies* as part of our contribution (cf. ①-②).

### 4.1. Modelling preference-based and context-based dependencies

An IUR is understood as a user-defined request referring to other users. Thus, when specifying an IUR, both the set of participating users and the service object or service class related with that IUR need to be determined. Further, a particular positive versus negative value is associated with the realisation of that IUR. We distinguish four basic types of IUR, regarding the two dimensions *relation* and *time* (cf. Table 1).

**Table 1.** Categorisation of IUR subject to the dimensions ‘relation’ and ‘time’.

		*Relation*	
		Complementary	Conflicting
***Time***	**Mutual** (time-independent)	*Complementary mutual usage* A user requests to use the same service object(s)/service class(es) **together** with one or more other users. A **positive** value is associated with this IUR.	*Conflicting mutual usage* A user requests **not** to use the same service object(s)/service class(es) **together** with one or more other users. A **negative** value is associated with this IUR.
	**Simultaneous** (time-dependent)	*Complementary simultaneous usage* A user requests to use and thus to start the same service object(s)/service class(es) **together** with one or more other users **at the same time**. A **positive** value is associated with this IUR.	*Conflicting simultaneous usage* A user requests **not** to use the same service object(s)/service class(es) **together** with one or more other users **at any moment in time**. A **negative** value is associated with this IUR.

Initially, an IUR refers to a certain single service object or a certain service class. Since an IUR concerns more than one user, preference-based dependencies exist among different users’ service compositions, which need to be taken into consideration when determining their utility. Further, simultaneous IUR additionally lead to dependencies of temporal nature, which are considered in Section 4.2 in detail.

When addressing context information in multi-user processes, we must account for the fact that CA attributes exist which refer to more than one user. A common example would be group discounts that will only be attained if a certain number of users will select the corresponding service object. Apart from that, CA attributes can also be time dependent like business hours. Accordingly, in Table 2 we distinguish four types of CA attributes, where each type represents a different kind of context-based dependency. Existing approaches merely address the single user-column of the table, which means they consider context-based dependencies and partially temporal-based dependencies for CA attributes referring to the service composition of a single user.

**Table 2.** Categorisation of CA attributes and dependencies subject to the dimensions ‘number of users’ and ‘time’.

		*CA Attributes with Relation to*	
		Single User	Multi User
***Time***	**Time-independent**	CA attributes resulting in dependencies **within** one user’s **service composition** e.g. distance, time-independent discount on service object A + B, favourite scores,*^8^* etc.	CA attributes resulting in dependencies **among** different users’ **service compositions** *e.g. time-independent group discount, etc.*
	**Time-dependent**	CA attributes resulting in dependencies **within** one user’s **service composition** **temporal-based** dependencies *e.g. availability/price of services objects dependent on daytime*	CA attributes resulting in dependencies **among** different users’ **service compositions** **temporal-based** dependencies *e.g. time-dependent group discount, etc.*

After systematising preference-based and context-based dependencies, we now model them formally. We first focus on preference-based dependencies resulting from IUR: In traditional single-user service selection, a user usually specifies her/his target weights and requirements regarding the NFP (cf. e.g. Alrifai et al., 2012; Yu et al., 2007; Zeng et al., 2004). When taking IUR into account, each user a∈A additionally has the possibility to specify a set of different IUR $E_{a}^{IUR}$. In doing so, a user a defines for each IUR $e\in E_{a}^{IUR}$ the set of participating users $A_{e}^{IUR}$, for each participating user the associated service object/service class (which results in the set $X_{e}^{IUR}$), and whether that IUR is of the *mutual* (time-independent) or *simultaneous* (time-dependent) type. Furthermore, the user sets a particular request value $q_{e}^{IUR}$ which is positive in the *complementary* case and negative in the *conflicting* case. This value corresponds to how important the user assesses the realisation of that IUR compared to other IUR she/he specified. To represent the importance of IUR, the user may also specify a target weight $w_{a}^{IUR}$. In that way, we consider IUR as regular attribute IUR∈N, more precisely as element of the subset of CA attributes O. As a consequence, for each IUR a utility value can be obtained through normalising and weighting the request value $q_{e}^{IUR}$ by means of the same utility function applied on the NCA and CA attributes of the selection problem as described in Section 3.3. Here, we differentiate the utility values ${\hat{U}}_{e}^{IUR}$ for mutual (time-independent) IUR and ${\bar{\overset{ˉ}{U}}}_{e}^{IUR}$ for simultaneous (time-dependent) IUR where the utility values can be positive or negative subject to the inherent case (complementary or conflicting).

Second, context-based dependencies resulting from CA attributes could be modelled in a similar way. In detail, we break down the dependencies caused by a CA attribute ∝∈O for each user a∈A into a set of single dependencies $E_{a}^{\propto }$. Furthermore, each dependency $e\in E_{a}^{\propto }$ encompasses a set of service objects $X_{e}^{\propto }$ which belong together in terms of utility or feasibility determination, for instance, the set of service objects which need to be selected to realise a certain group discount. In case the dependency e refers to utility determination regarding the set of service objects $X_{e}^{\propto }$, the corresponding utility associated with the CA attribute is obtained based on the quantified value $q_{e}^{\propto }$ of the related context information by applying the utility function. Here, we also differentiate between a utility value ${\hat{U}}_{e}^{\propto }$ for time-independent CA attributes and a utility value ${\bar{\overset{ˉ}{U}}}_{e}^{\propto }$ for time-dependent CA attributes. To additionally consider the case of feasibility determination (e.g. business hours), we further consider the set $F_{e}^{\propto }$. This set is required to determine the feasibility of the service objects $X_{e}^{\propto }$, otherwise $F_{e}^{\propto }=\emptyset$. Moreover, the set $A_{e}^{\propto }$ is specified as the subset $A_{e}^{\propto }\subseteq A$ of users that are associated with that dependency e. In case the corresponding CA attribute ∝ is referring only to a single user, $\left|A_{e}^{\propto }\right|=1$ holds for each $e\in E_{a}^{\propto }$ (e.g. business hours), otherwise $\left|A_{e}^{\propto }\right|>1$ (e.g. group discounts).

Based on that, a single dependency $e\in E_{a}^{\propto }$ describing IUR as well as CA attributes is represented by the following 5-tuple (cf. Appendix A for the used notation):

(3) $$e=\left({\hat{U}}_{e}^{\propto },{\bar{\overset{ˉ}{U}}}_{e}^{\propto },F_{e}^{\propto },A_{e}^{\propto },X_{e}^{\propto }\right)$$

In general, the utility value ${\hat{U}}_{e}^{\propto }$ is distinct from 0 if the corresponding IUR or CA attribute is time independent, and the utility value ${\bar{\overset{ˉ}{U}}}_{e}^{\propto }$ is distinct from 0 if the corresponding IUR or CA attribute is time dependent. However, they are both equal 0 and $F_{e}^{\propto }\neq \emptyset$ if e only refers to feasibility determination. Note, $X_{e}^{\propto }$ contains one or more decision variables $x_{a_{ij}}$ for each user $a\in A_{e}^{\propto }$, where $x_{a_{ij}}$ is the binary decision variable corresponding to the service object $s_{ij}$ for user a, and which is used in the optimisation models proposed later on. That is, $x_{a_{ij}}$ is 1 if the corresponding service object $s_{ij}$ is selected for user a, and 0 if not. Further, by breaking down the dependencies of an IUR or CA attribute, it can be assured that the utility determined regarding a single dependency is definite, which means the associated positive or negative utility is realised if – and only if – all service objects in $X_{e}^{\propto }$ are part of the solution. The same applies for feasibility determination.

In conclusion, when taking CA attributes and IUR into account, the utility and feasibility determination of a service object or set of service objects requires the consideration of other service objects, too. However, we are able to model the resulting context-based and preference-based dependencies through sets of dependencies $E_{a}^{\propto }$ (with ∝∈O, where IUR∈O and $F_{e}^{\propto }=\emptyset$ for all preference-based dependencies) where the values of ${\hat{U}}_{e}^{\propto }$ and ${\bar{\overset{ˉ}{U}}}_{e}^{\propto }$ indicate whether the utility determination of the dependency is of temporal nature or not, and the set $F_{e}^{\propto }$ whether the feasibility determination is time dependent or not.

### 4.2. Modelling temporal-based dependencies

The consideration of simultaneous IUR and time-dependent CA attributes also leads to dependencies of temporal nature (cf. Tables 1 and 2). More precisely, the utility or feasibility of a service object/set of service objects depends not only on the selection of other (preceding or succeeding) service objects but also on the exact point in time of their intended usages – and thus on the duration of all preceding service objects of the service composition. In this context, the possibility *to wait* for the users instead of switching (or being forced to switch) to another, less favoured service object needs to be considered as well. When using waiting time as buffer (if necessary or if it creates higher utility), we need to take into account the service compositions of all users.

Thereby, a concept for modelling and integrating waiting times in an optimisation model is required. Regarding simultaneous IUR, there needs to be the possibility to wait for a user in order to realise a positive utility associated with a complementary simultaneous IUR or to avoid a negative utility associated with the realisation of a conflicting simultaneous IUR. In the case of time-dependent CA attributes, the delay achieved through waiting may enable an infeasible service object to become feasible (e.g. business hours) or may lead to a higher utility (e.g. time-dependent discounts), despite a decrease in utility which may be associated with the waiting time.

**Figure 2.**
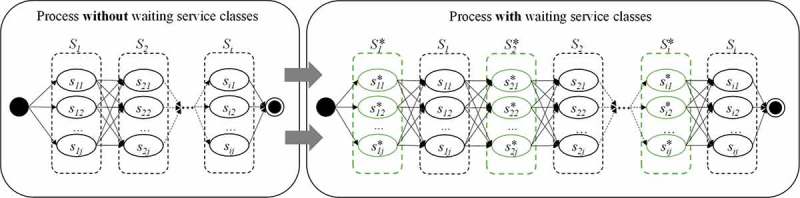
Illustration of a process without and with waiting service classes.

To enable this, we introduce the additional NCA attribute waiting time WT (with $WT\in N^{-}$) similar to duration. Moreover, to avoid an increasing complexity when modelling the optimisation problem, we propose special waiting service classes $S_{i}^{*}$ right in front of each regular service class $S_{i}$ as an alternative for a user to wait right between two succeeding regular service classes. Each waiting service class encompasses a set of waiting services where each waiting service $s_{ij}^{*}\in S_{i}^{*}$ is only described by the NCA attribute WT (i.e. all other NFP values are 0) to represent different manifestations of waiting time within one waiting service class. This allows us to model the time consumed by waiting as well as the resulting loss of utility caused by waiting. By placing a waiting service class right before each regular service class as illustrated in Figure 2, the service object selected in the regular service class can be delayed by the amount of WT related to the selected waiting service.

As an example, let us consider a user $a_{1}$ requesting to use service object $s_{2\;2}$ simultaneously together with user $a_{2}$ (i.e. complementary simultaneous IUR), which implies that for realising the utility associated with this IUR, both users must use service object $s_{2\;2}$ at the same point in time. Therefore, potential waiting times depend on the duration $q_{ij}^{Dur}$ of the service objects both users have already accomplished so far (here: service objects selected in service class $S_{1}$). As a result, three possible alternatives can be distinguished:

- Waiting is not necessary (e.g. if the aggregated duration of the selected service compositions till using service object $s_{2\;2}$ is the same for both users)
- Waiting time is proposed for one of the two users (e.g. if the aggregated duration of the selected service compositions until using service object $s_{2\;2}$ is different for the users)
- Waiting is dispensable (e.g. the IUR and the associated utility will not be realised)

To decide which alternative is the most beneficial, an optimisation model must evaluate if the additional utility realised by the IUR outweighs the loss of utility caused by waiting, which depends upon the amount of waiting time necessary. Considering the entire service composition, this can also lead to the selection of alternative preceding and succeeding service objects. To enable the determination of the right amount of waiting time $q_{ij}^{WT}$, we propose to model attributes representing ‘time’ (e.g. duration) as discrete, such that $q_{ij}^{WT},\;q_{ij}^{Dur}\in \left\{k*c|k\in N_{0}\right\}$, with $c\in R^{+}$. Thus, each waiting service $s_{ij}^{*}\in S_{i}^{*}$ represents a different discrete manifestation of waiting time (e.g. discrete steps of 15 min). We argue that this seems appropriate for most service selection problems at planning time as the parameter c can be adjusted to every purpose or need.

## 5. Optimisation models for a stateless versus stateful representation

To incorporate preference-based, context-based and temporal-based dependencies in an optimisation-based approach, a stateful or a stateless representation can be applied (cf. ③). In the latter case, dependencies can only be regarded directly within the scope of the optimisation model itself, whereas with a stateful representation the consideration of dependencies could also take place by explicitly modelling a state space in combination with the determination of utility and feasibility. Although both forms of representation are feasible, there are differences regarding criteria like model complexity and computational complexity (cf. Section 6.2 Performance Evaluation).

### 5.1. Stateless representation

In the stateless representation, the multi-user context-aware service selection problem can be formulated as knapsack problem where the purpose of the corresponding optimisation model lies in determining the optimal service compositions for all users. Thereby, we propose to use the decision variables $x_{a_{ij}}$ for each user a∈A and every (regular and waiting) service object $s_{ij}$ of the underlying process. Each decision variable $x_{a_{ij}}$ is associated with a utility value $U_{a_{ij}}$ which could possibly be different for each user – subject to the user-defined target weights $w_{a}^{\propto }$ regarding the NFP. Here, $U_{a_{ij}}$ only represents the utility value for the NCA attributes concerning user a and service object $s_{ij}$. For utility determination of time-independent and time-dependent CA attributes and IUR, we apply the proposed modelling in terms of the utility values ${\hat{U}}_{e}^{\propto }$ and ${\bar{\overset{ˉ}{U}}}_{e}^{\propto }$ and the corresponding set of service objects $X_{e}^{\propto }$. In line with this, we divide our set O of CA attributes and IUR in elements $\hat{O}$ which require time-independent utility determination and those elements $\overset{ˉ}{O}$ which require time-dependent utility determination. Thus, for the stateless case, we can formulate our optimisation model, which is non-linear, as follows:

(4) $$\begin{array}{c} \underset{x_{a_{ij}};s_{e}^{\propto }}{\max}\underset{a\in A}{\sum }\overset{I}{\underset{i=1}{\sum }}\underset{s_{ij}\in S_{i}}{\sum }U_{a_{ij}}x_{a_{ij}}+\underset{a\in A}{\sum }\underset{\propto \in \hat{O}}{\sum }\underset{e\in E_{a}^{\propto }}{\sum }{\hat{U}}_{e}^{\propto }\underset{x_{a_{ij}}\in X_{e}^{\propto }}{\prod }x_{a_{ij}}\\ +\underset{a\in A}{\sum }\underset{\propto \in \bar{\overset{ˉ}{O}}}{\sum }\underset{e\in E_{a}^{\propto }}{\sum }{\bar{\overset{ˉ}{U}}}_{e}^{\propto }s_{e}^{\propto }\underset{x_{a_{ij}}\in X_{e}^{\propto }}{\prod }x_{a_{ij}} \end{array}$$

(5) $$s.t.\;\overset{I}{\underset{i=1}{\sum }}\underset{s_{ij}\in S_{i}}{\sum }q_{ij}^{\propto }x_{a_{ij}}\leq Q_{a}^{\propto }\;\;\;\forall \propto \in M;\forall a\in A$$

(6) $$\underset{e\in E_{a}^{\propto }}{\sum }q_{e}^{\propto }\underset{x_{a_{ij}}\in X_{e}^{\propto }}{\prod }x_{a_{ij}}\leq \;Q_{a}^{\propto }\;\;\;\forall \propto \in O;\forall a\in A$$

(7) $$\underset{s_{ij}\in S_{i}}{\sum }x_{a_{ij}}=1\forall i=1\;to\;I;\forall \;a\in A;with\;x_{a_{ij}}\in \left\{0,1\right\};\;s_{e}^{\propto }\in \left\{0,1\right\}$$

The objective function (4) determines the accumulated maximum utility over all users a∈A, all service classes $S_{i}$ and all service objects $s_{ij}$ by taking into account the binary decision variables $x_{a_{ij}}$ and $s_{e}^{\propto }$ ($x_{a_{ij}}=1$ indicates that service object $s_{ij}$ is selected for user a, $x_{a_{ij}}=0$ that is not). The first summand of the function $\underset{a\in A}{\sum }\overset{I}{\underset{i=1}{\sum }}\underset{s_{ij}\in S_{i}}{\sum }U_{a_{ij}}x_{a_{ij}}$ refers to utility determination regarding NCA attributes where no dependencies need to be considered. The second summand represents time-independent utility determination, for example, for mutual IUR. Here, the associated (positive or negative) utility ${\hat{U}}_{e}^{\propto }$ is realised if the product $\underset{x_{a_{ij}}\in X_{e}^{\propto }}{\prod }x_{a_{ij}}$ is 1, which is only the case if all service objects given in $X_{e}^{\propto }$ are actually selected. In terms of time-dependent utility determination, additional constraints are required to enable the consideration of temporal-based dependencies. This is achieved by the third summand through relating the product of the decision variables $x_{a_{ij}}$ and the associated utility ${\bar{\overset{ˉ}{U}}}_{e}^{\propto }$ to an indicator variable $s_{e}^{\propto }$, that is 1 if the corresponding constraints hold and 0 if not. The formulation of the constraints depends upon the specific temporal relationship that needs to be satisfied to realise the utility.

In terms of feasibility determination, constraints (5) and (6) consider the global end-to-end constraints for NCA and CA attributes defined by the users. The consideration of feasibility determination referring to any dependencies between service objects is also achieved by adding constraints to the optimisation model. Similar to the time-dependent utility determination, their concrete formulation depends upon the set $F_{e}^{\propto }$. To hold the (standard) condition that for each user a∈A and for every service class $S_{i}$ exactly one service object must be selected, constraints (7) have also be part of our optimisation model.

Appendix B shows the stateless optimisation model and additional constraints required for time-dependent utility determination in terms of the integration of complementary and conflicting simultaneous IUR.

### 5.2. Stateful representation

**Figure 3.**
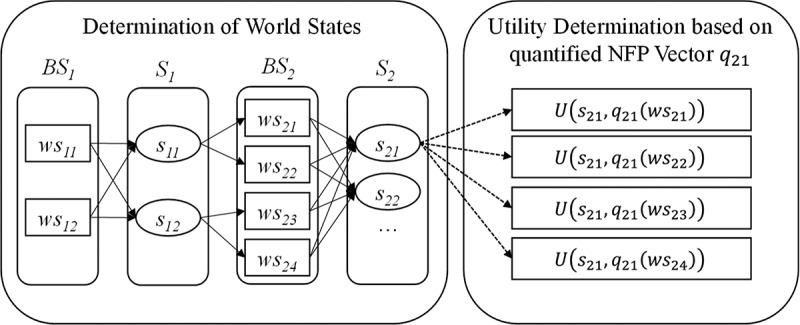
Illustration of utility determination with respect to world states determination (cf. Heinrich & Lewerenz, 2015).

For our stateful approach, we base upon the concept of belief and world states (cf. Ghallab et al., 2004): Accordingly, a state space consists of one belief state $BS_{i}$ for each action of the process where each belief state encompasses a set of belief state tuples $bst_{ik}$ (with i referring to the corresponding service class $S_{i}$ and k as the number of the tuple). Further, each world state $ws_{ik}\subseteq bst_{ik}$ holds exactly one state variable $v\left(bst_{ik}\right)$ for each context information and its corresponding value. Finally, $BS_{1}$ represents the initial state of the process and $BS_{I+1}$ the goal state, accordingly. The utility of a particular service object is then determined in respect of a certain world state, which means based on its quantified non-context and context information as illustrated in Figure 3. These generated state-service combinations (i.e. the state-service space) could then be used within an optimisation model to determine the best service composition for each user with regard to context information. In terms of feasibility determination referring to context-based dependencies, world states and service objects which are not feasible regarding their determined values will not be considered any further.

The main benefit of such a stateful representation is that the size of the state space for a user remains mostly constant regardless the number of different types of context information considered. But so far, in existing approaches only context-based dependencies in terms of single-user service selection are considered. This means, we need to extend those approaches by both multiple users (cf. ①) and possible waiting times (cf. ②). We propose therefore to determine in a first step the state space for each user a∈A separately because each user may have her/his individual initial context (i.e. initial state $BS_{a_{1}}$), and determine then context-based and temporal-based dependencies that exist within the user’s own service composition. As a result, each user a∈A has its own state space consisting of belief states $BS_{a_{i}}$, belief state tuples $bst_{a_{ik}}$ and world state tuples $ws_{a_{ik}}$. Since waiting time and waiting service classes could be modelled as NCA attribute and regular service classes, they also result in belief states $BS_{a_{i}}$. To determine the values of the state variables $v\left(bst_{a_{ik}}\right)\in ws_{a_{ik}}$, an existing state-transition algorithm (e.g. Heinrich & Lewerenz, 2015) needs to be extended: As the value of each state variable depends upon the corresponding service object and – subject to the type of CA attribute – also on the preceding world state, the state transition for each variable $v\left(bst_{a_{ik}}\right)\in ws_{a_{ik}}$ could be defined as $v\left(bst_{a_{i^{\prime }k}}\right)\leftarrow \Phi \left(q_{a_{ij}}^{\propto },v\left(bst_{a_{ik}}\right)\right)$.^4^

However, dependencies resulting from IUR and CA attributes that exist among different users’ service compositions require the determination of a joint state space for all participating users. But the implicit modelling of all possible service combinations regarding all users seems not a very promising approach in terms of computational complexity. Therefore, we propose a different way: For each dependency $e\in E_{a}^{\propto }$ with $\left|A_{e}^{\propto }\right|>1$, we determine the set of associated world states in the created state spaces of the users $a\in A_{e}^{\propto }$. When considering time-dependent utility or feasibility determination (e.g. simultaneous IUR), there could exist more than one of such a set of world states, for instance, referring to different manifestations of daytime $v^{Time}\left(bst_{a_{ik}}\right)$. These sets of world states form the set $Z_{e}^{\propto }$, which is then linked to a new world state $ws_{e}^{\propto }$ addressing the dependency e.

In the optimisation model, the optimal solution over all users could then be calculated based on the determined state-service combinations of all users. In this regard, the objective function is formulated as follows:

(8) $$\begin{array}{rl} & \underset{x_{a_{ij}};\;y_{a_{ik}};y_{e}^{\propto }}{\max}\underset{a\in A}{\sum }\overset{I}{\underset{i=1}{\sum }}\underset{s_{ij}\in S_{i}}{\sum }\underset{\begin{array}{c} ws_{a_{ik}}\\ \in BS_{a_{i}} \end{array}}{\sum }U\left(s_{ij},\;q_{a_{ij}}\left(ws_{a_{ik}}\right)\right)*x_{a_{ij}}*y_{a_{ik}}\\ & +\underset{a\in A}{\sum }\underset{\propto \in O}{\sum }\underset{\left\{\begin{array}{c} e\in E_{a}^{\propto }|\\ \left|A_{e}^{\propto }\right|>1\wedge \\ \left({\hat{U}}_{e}^{\propto }\neq 0\vee {\bar{\bar{U}}}_{e}^{\propto }\neq 0\right) \end{array}\right\}}{\sum }U\left(q_{e}^{\propto }\left(ws_{e}^{\propto }\right)\right)*y_{e}^{\propto } \end{array}$$

Similar to the stateless representation, the accumulated maximum utility is achieved by setting the corresponding binary decision variables $x_{a_{ij}}$, $y_{a_{ik}}$ and $y_{e}^{\propto }$. Here, $y_{a_{ik}}$ indicates whether the world state $ws_{a_{ik}}$ for user a is selected or not, and, likewise, $y_{e}^{\propto }$ indicates whether the world state $ws_{e}^{\propto }$ related to a dependency e is selected or not.

The first summand in the objective function (8) encompasses utility determination for all NCA and CA attributes referring to a single user, which means context-based and temporal-based dependencies existing within a user’s service composition are considered. Generally, for each service class, only one service object $s_{ij}$ and for each belief state only one world state $ws_{a_{ik}}$ is selectable (see complete model in Appendix C). Further, the second summand deals with utility determination for dependencies existing among different users’ service compositions and hence for IUR and CA attributes referring to multiple users. More precisely, $U\left(q_{e}^{\propto }\left(ws_{e}^{\propto }\right)\right)$ corresponds to the utility values ${\hat{U}}_{e}^{\propto }$ and ${\bar{\overset{ˉ}{U}}}_{e}^{\propto }$ and is realised if $y_{e}^{\propto }=1$, which means if the state $ws_{e}^{\propto }$ is selected. The required link of $y_{e}^{\propto }$ (and $ws_{e}^{\propto }$) to the associated service objects $x_{a_{ij}}\in X_{e}^{\propto }$ and the determined world state sets $Z_{e}^{\propto }$ is achieved through the following constraint:

(9) $$y_{e}^{\propto }-\underset{Z_{e_{k}}^{\propto }\in Z_{e}^{\propto }}{\sum }\underset{\left\{\begin{array}{c} a\in A_{e}^{\propto }|\\ x_{a_{ij}}\in X_{e}^{\propto } \end{array}\right\}}{\prod }x_{a_{ij}}\underset{ws_{a_{ik}}\in Z_{e_{k}}^{\propto }}{\sum }y_{a_{ik}}=0$$

$$\forall \propto \in O;\forall a\in A;\forall \begin{array}{c} e\in E_{a}^{\propto }\;with\;\left|A_{e}^{\propto }\right|>1\wedge \left({\hat{U}}_{e}^{\propto }\neq 0\vee {\bar{\overset{ˉ}{U}}}_{e}^{\propto }\neq 0\right) \end{array}$$

By this, dependencies resulting from mutual and simultaneous IUR as well as CA attributes referring to multiple users could be integrated straightforwardly in a stateful representation. The complete optimisation model also encompasses both constraints for considering the users’ requirements regarding the NCA and CA attributes and constraints for feasibility determination dealing with dependencies among multiple users’ service compositions. As a result, preference-based, context-based and temporal-based dependencies resulting from IUR and CA attributes could be considered upon the state spaces of the users in combination with the optimisation model.

## 6. Evaluation

In this section, we provide an evaluation of our approach. In detail, we want to show how our approach could provide decision support, which we will evaluate based on the scenario introduced in Section 2 in terms of the criterion *efficacy*. To analyse the computation time of the stateless and stateful model with respect to different multi-user context-aware service selection problems, we additionally evaluate our approach regarding the criterion *performance*. By this, the design of our evaluation follows the compositional styles *demonstration* and *simulation- and metric-based benchmarking of artefacts* (cf. Prat, Comyn-Wattiau, & Akoka, 2015). We use integer programming (Nemhauser & Wolsey, 1988) to find the optimal solution for both optimisation models. For this purpose, our presented non-linear optimisation models are transformed into linear ones, which are used throughout the evaluation.

To examine whether our stateless and stateful models provide the optimal service compositions and are consistent to each other, we implemented the linearised versions of the two models in Java and used the mathematical programming solver Gurobi Optimiser^5^ for solving them. To ensure a correct implementation, we conducted intensive testing of the source code (i.e. manual analysis by other persons than the programmers, unit tests, JUnit regression tests, runs with extreme values). We then compared the optimal service compositions obtained from our stateless and stateful optimisation models with an exhaustive enumeration (for small problem sizes). In this regard, we analysed the results of over 15,000 randomly generated multi-user context-aware service selection problems (with a maximum problem size related to 16,777,216 possible service compositions). As the solutions were invariably the same for the enumeration, the stateless and the stateful model, we are convinced that our optimisation models are consistent and provide the correct solution.

### 6.1. Efficacy

We analyse the efficacy of our approach in terms of the real-world scenario described in Section 2: A city day trip to Munich, Germany, by three users that encompasses eight different activities (visiting a museum, having lunch, etc.). Using *TripAdvisor* ^6^ and *Google Places* ^7^, we determine feasible service objects and their NFP (price, GPS location, business hours, duration) for each of the eight activities, where service objects with no fixed duration are modelled multiple times – each with a different possible manifestation of duration (e.g. a visit of a museum may last 60 min, 90 min, etc.). By this, we consider a process which can be realised by over 2.9 billion possible service compositions per user.

To demonstrate the efficacy of our approach, we compare the solution of i) an existing single user context-aware service selection approach (i.e. the approach presented by Heinrich and Lewerenz (2015) for each user separately) to the solution of ii) our multi-user context-aware approach (regardless of whether using the stateless or stateful model here as they both provide the same solution). Thereby, we consider – by utilising the information gathered about the available service objects – the NCA attributes *duration* and *price* and the CA attributes *distance* (between two succeeding service objects subject to their GPS location) and *business hours*. Moreover, to get realistic initial contexts as well as target weights and requirements regarding these NCA and CA attributes in our scenario, we conducted a small laboratory experiment with three graduated students named *Pam, Marc* and *Dan* (Table 3). Additionally, we asked each of the students to define four *IUR* (one of each type) which are listed in Table 4. Further, we consider *group discounts* and the NCA attribute *waiting time*. The regarded discrete values of duration and waiting time range from 0 to 120 in steps of 15 min.

**Table 3.** Parameter settings retrieved by the laboratory experiment.

Parameter	Pam	Marc	Dan
NCA duration	target weight: 0.1 constraint: 650 min	target weight: 0.05 constraint: 650 min	target weight: 0.1 constraint: 600 min
NCA waiting time	target weight: 0.1 constraint: 30 min	target weight: 0.2 constraint: 20 min	target weight: 0.2 constraint: 80 min
NCA price	target weight: 0.5 constraint: 80 €	target weight: 0.05 constraint: 90 €	target weight: 0.2 constraint: 80 €
CA distance	target weight: 0.1 constraint: 15 km initial context: P + R Froettmaning	target weight: 0.3 constraint: 45 km initial context: Main station	target weight: 0.4 constraint: 10 km initial context: Karlsplatz Stachus
CA business hours	initial context: 11:45 am	initial context: 11:30 am	initial context: 11:30 am
IUR	target weight: 0.2	target weight: 0.4	target weight: 0.1

**Table 4.** IUR specified for city day trip.

Defining User	Type of IUR	Referred Users	Action		Service Object		Utility
			ID	Name	ID	Name	
Pam	compl. simultaneous	2, 3	2	Lunch	3	Bavarese	+0.6
Pam	compl. mutual	2	3	Sight	0	Kaufinger- und Neuhauser Strasse	+0.04
Pam	confl. simultaneous	3	4	Active Life	4	Botanischer Garten Muenchen	−0.2
Pam	confl. mutual	2, 3	8	Culture	5	Deutsches Theater Muenchen	−0.14
Marc	compl. simultaneous	1, 3	7	Nightlife	3	CA-BA-LU	+0.08
Marc	compl. mutual	1, 3	4	Active Life	8	Froettmaninger Berg	+0.2
Marc	confl. simultaneous	1	3	Sight	4	Muenchner Freiheit	−0.04
Marc	confl. mutual	1	3	Sight	7	Maximilianeum – Bayerischer Landtag	−0.36
Dan	compl. simultaneous	2	5	Café	-	-	+0.08
Dan	compl. mutual	1	2	Lunch	7	Restaurant Al Paladino	+0.04
Dan	confl. simultaneous	1	7	Nightlife	9	Loretta	−0.06
Dan	confl. mutual	1	7	Nightlife	0	Ryans Muddy Boot	−0.02

Given this setting, we compare the results of both approaches i) and ii), which means, the optimal service composition for each user and the corresponding NFP values (cf. Table 5): considering service class *5) Café* and the users Marc and Dan in approach ii), we recognise that – in contrast to i) – for both users the same service object $s_{5\;27}$ (referring to a café named ‘Puck’) is selected. This can be directly ascribed to the realisation of the complementary simultaneous IUR ‘Dan requests to take a coffee together with Marc regardless which café’ (cf. ①), but which also requires Dan to wait 45 min in total. However, for Dan the realisation of that IUR is still of higher value than waiting 45 min, which means, the positive utility ${\bar{\bar{U}}}_{e}=0.08$ Dan associated with that IUR is able to compensate the loss of utility resulting from waiting. Another realised complementary but mutual IUR is ‘Pam requests to visit the sight “Kaufinger and Neuhauser Street” with Marc’ (service object $s_{3\;10}$). On the other side, the conflicting mutual IUR ‘Pam requests not to go all together to the “German Theatre Munich”’ (service class *8) Culture*) is not realised as none of the three users visits that theatre. Consequently, the utility of Pam’s overall service composition is not decreased by the associated negative utility ${\hat{U}}_{e}=-0.06$. Furthermore, because of a group discount of 2.00 € each in approach ii) both Marc and Dan visit the museum ‘Pinakothek of Modern Art’ ($s_{1\;10}$) and thus achieve a lower price (resulting in a higher utility) compared to i). To be able to go to the favoured dinner restaurant with respect to its business hours, in approach i) Dan needs to spend 15 min longer in one of the previous actions since the option to wait is not considered. In approach ii) instead, he waits 15 min as he prefers waiting over spending more time than favoured in one of the other actions (cf. ②). This analysis illustrates the efficacy when considering ①-② in a multi-user context-aware service selection which is also supported by the discussion of the results with the three graduated students participating in the scenario.

**Table 5.** Solution of i) existing approaches versus ii) multi-user context-aware approach for a city day trip scenario.

	User	Optimal Service Composition	Duration (min)	Wai-ting Time (min)	Dis-tance (km)	Price (€)	Group Dis-count (€)	Rea-lised IUR
i) Existing Approaches	**Pam**	*s_1 18_, s_2 20_, s_3 8_, s_5 26_, s_6 16_, s_7 11_*	540	./.	12.801	60.00	./.	./.
	**Marc**	*s_1 11_, s_2 20_, s_3 8_, s_5 26_, s_6 16_, s_7 19_*	540	./.	3.820	65.00	./.	./.
	**Dan**	*s_1 1_, s_2 28_, s_4 27_, s_5 7_, s_6 4_, s_7 1_*	450	./.	5.451	60.00	./.	./.
ii) Multi-User Context-Aware Approach	**Pam**	*s_1 18_, s_2 20_, s_3 10_, s_5 26_, s_6 10_, s_7 11_*	555	0	12.834	60.00	0.00	1
	**Marc**	*s_1 10_, s_2 28_, s_3 10_, s_5 27_, s_6 14_, s_7 11_*	555	0	6.690	58.00	2.00	0
	**Dan**	*s_1 10_, s_2 8_, s_4 17_, s_5 27_, s_6 4_, s_7 1_*	450	45	5.503	58.00	2.00	1

### 6.2. Performance

In this section, we analyse the stateless and stateful models with respect to their performance, which means, the computation time needed by them for solving multi-user context-aware service selection problems. With evaluating a NP-hard problem (Abu-Khzam, Bazgan, Haddad, & Sikora, 2015) and an approach determining the optimal solution, we expect an over-proportional growth in computation time with increasing problem size (Nemhauser & Wolsey, 1988). Computation time in the context of service selection usually depends on several parameters (Alrifai & Risse, 2009). The influence of parameters referring to traditional single-user service selection, such as *number of service classes, number of service objects, number of considered NFP*, and so on, has already been studied thoroughly in literature. Thus, we focus on parameters related to our contribution ①-③: i) the *number of users*, ii) the *number of IUR*, iii) the *number of CA attributes*, and iv) the *number of waiting services* per waiting service class.

For our evaluation, we conduct a simulation experiment and an artificial dataset with randomly generated values. Our *initial problem size* encompasses four regular service classes á six service objects and – to consider waiting time – four waiting service classes á five waiting services per class, where waiting time is increased from 0 to 60 in steps of 15 time units. Further, the problem consists of three users, twelve IUR (i.e. four IUR per user, one of each type), three NCA attributes (duration, waiting time and price) and one CA attribute (distance type as representative for other CA attributes). Appendix D summarises the basic evaluation configuration. Founded on this basic configuration, we use four different scenarios corresponding to the four analysed parameters. In each scenario, one parameter is altered while all other parameters are kept constant as defined in the basic evaluation configuration (i.e. *ceteris paribus*):

- The number of users is increased from 2 to 10 in steps of 1
- The number of IUR per User is increased from 2 to 10 in steps of 2 (1 complementary and 1 conflicting)
  - in terms of mutual IUR (in the absence of simultaneous IUR)
  - in terms of simultaneous IUR (in the absence of mutual IUR)
- The number of distance type CA attributes is increased from 1 to 10 in steps of 1
- The number of waiting services per class is increased from 3 to 10 in steps of 1

**Figure 4.**
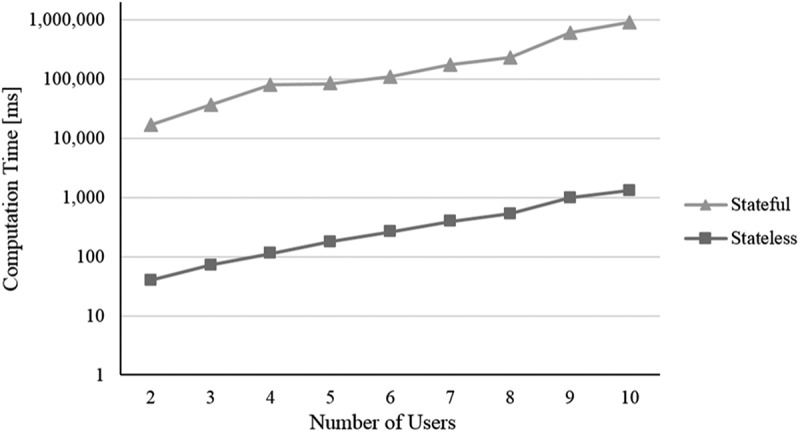
Scenario i).

**Figure 5.**
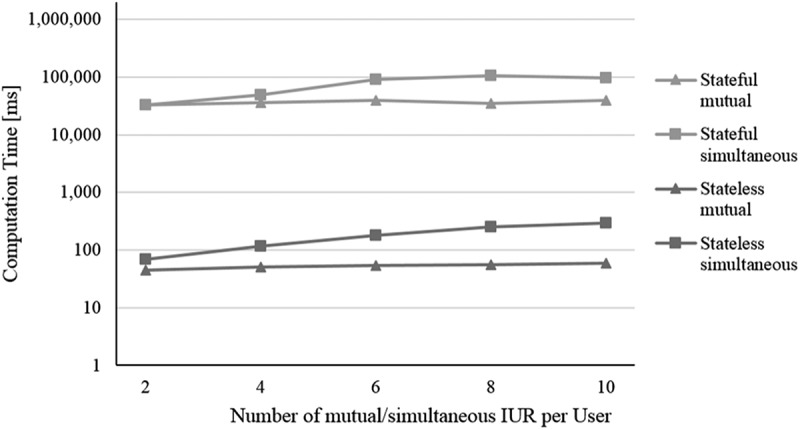
Scenario ii).

**Figure 6.**
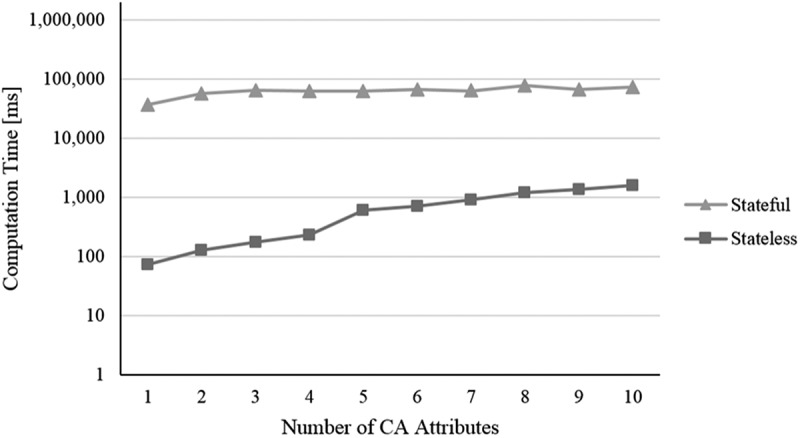
Scenario iii).

**Figure 7.**
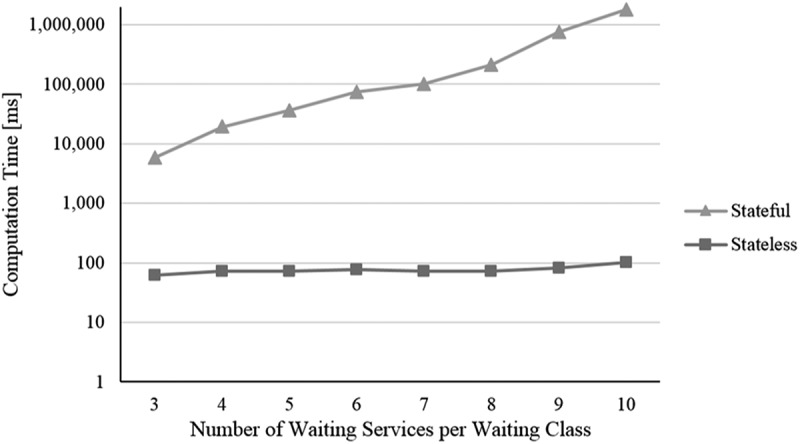
Scenario iv).

For all simulation runs, we use a machine with an Intel Xeon E5-2470 v2 processor with 2.40 GHz, 32 GB RAM, Win7 64bit, Java 1.8, and the mathematical solver Gurobi Optimiser 6.5. We conduct for each setting regarding the four scenarios i) to iv) 200 simulation runs and determine the average computation time (measured in milliseconds [ms]). To be able to compare the results of both optimisation models, the measured computation time encompasses not only the time Gurobi Optimiser needs for solving a model but also the time required for building a model, which includes the state space creation in terms of the stateful representation. In the following, the results are presented (cf. Figures 4–7):

When increasing the *i) number of users*, not only the number of variables and constraints regarding the additional users increase but also the number of dependencies resulting from IUR. As shown in Figure 4, this leads for both models to a continuous increase in computation time. To analyse *ii) the influence of time-independent (mutual) as well as time-dependent (simultaneous) IUR*, we consider them in separate simulation runs (cf. Figure 5). In the case of mutual IUR, the stateless model as well as the stateful model show an apparent slighter increase in computation time compared to simultaneous IUR. This is because mutual IUR only have a minor effect on the number of additional variables and constraints of the optimisation models, whereas for simultaneous IUR also temporal-based dependencies need to be considered, which results in a higher number of constraints. As the state space of the stateful model mostly remains constant in size regardless of the number of considered CA attributes, we do expect the computation time staying pretty much the same for the stateful model when increasing the *iii) number of CA attributes*. As Figure 6 illustrates, this is supported by our simulation experiment. In contrast, the stateless model shows a greater increase in computation time, resulting from the higher number of variables and constraints that must be considered with each additional CA attribute and the corresponding context-based dependencies. When increasing the *iv) number of waiting services* per class from 3 to 10, an increase in computation time is only apparent for the stateful model (cf. Figure 7). This results from the fact that each waiting service increases the state space by adding a new manifestation of daytime and therefore leads to a (significantly) larger state space. The stateless model however seems much more robust here. Indeed, an additional experiment reveals an average computation time of only 348 ms for 150 waiting services per waiting class.

To sum up, considering our simulation experiment and scenarios, the performance of the stateless model is obviously much better than the stateful model. The reason is the high number of variables that need to be additionally considered through the creation of the state space. Furthermore, the stateful model appears to be more sensitive regarding the number of waiting services while the stateless model seems to be more sensitive regarding the number of CA attributes. In terms of the number of users and the number of IUR per user, both models show a rather similar change of computation time. As we do not aim to present a computation time optimised approach (e.g. a heuristic) but rather a first approach for a multi-user context-aware service selection at planning time, the computation times especially of the stateless model seem quite acceptable.

## 7. Discussion

**Figure 8.**
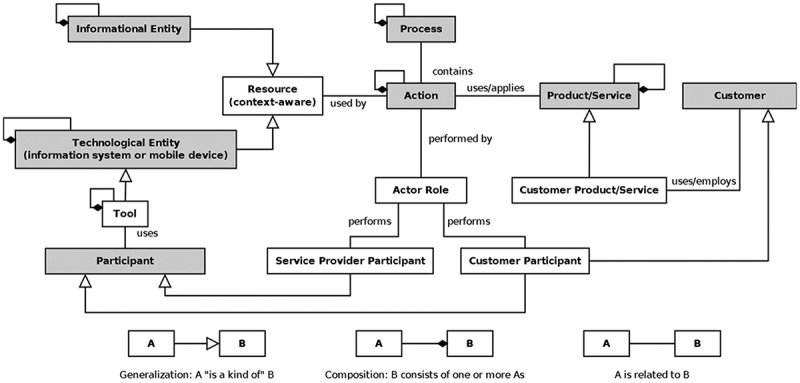
Excerpt of the meta model for a multi-user context-aware service system (based on Alter, 2012).

This section discusses theoretical as well as practical implications of our work. Starting with theoretical implications, the multi-user context-aware service selection scenarios described in the paper can also be understood in general as service systems (cf. Alter, 2012) – in terms of a context-aware interplay of stationary and mobile devices, services and users (Zaplata, Kunze, & Lamersdorf, 2009). In this regard, collaboration and contextualisation are part of service-dominant design which forms the basis for modern service systems (Alter, 2012; Böhmann, Leimeister, & Möslein, 2014; Edvardsson, Ng, Zhi Min, Firth, & Yi, 2011). Collaboration (in terms of co-creation and co-consumption) means that the value of a considered service is created by multiple users (Grönroos, 2011; Vargo & Lusch, 2004). In adoption of the meta model presented by Alter (2012), additional value can be created by a context-aware selection of informational entities (service objects) as resources to perform actions of processes in mobile environments as illustrated in Figure 8.

In this meta model, each customer resp. user may conduct his own actions and processes for which informational (in terms of service objects) and technological entities (e.g. mobile devices) need to be selected resp. used as required resources. In mobile environments, this selection is typically context aware. In addition, taking co-consumption in form of IUR into account means that the mutual/simultaneous conduction of some actions by more than one user from otherwise possibly different processes generates additional (positive or negative) value for the users. According to this, analysing and modelling a multi-user context-aware service selection is an actual instantiation of the theoretical construct of a service system, which was proven by this research in order to carefully and specifically evaluate the general construct.

In terms of practical implications, practitioners should be aware that there could be significant advances regarding the optimal service compositions when using the presented approach (cf. Section 6.1). This is not only due to the consideration of dependencies resulting from multiple users and context information. Indeed, by selecting and presenting the optimal service objects for each user regarding an entire service composition, it also addresses the problem of information overload (Zhang et al., 2009) a decision maker may often be confronted with in such situations. By this, we are confident that practitioners could substantially benefit from our work when selecting services (or service objects) for a context-aware process with multiple participating users. For example, we currently plan to validate our approach in an interesting use case together with a big German automotive company: We want to realise our approach in an application for mobile meeting coordination supported in an automated manner. The companies’ employees typically attend a lot of meetings every week while not all of these meetings are mandatory but valuable (in different levels) for the employees. However, due to the size of the company meeting, planning can be very challenging as the potential participants and meeting rooms are distributed over several facilities. Therefore, the distance (and thus the time) the participants need to cover to get to the location also needs to be considered. Here, we are convinced that an app implementing our approach can support the companies’ employees in determining the optimal time and location for a meeting.

## 8. Conclusion, limitations and further research

Within this work, we presented a multi-user service selection approach, which is to the best of our knowledge the first optimisation-based approach that takes multiple users and context information into account. In this regard, both optimisation models cope with preference-based, context-based and temporal-based dependencies. Existing approaches either focus on context information in terms of single-user service selection or hard restrictions in terms of multi-user service selection (e.g. capacity limits) and neglect potential waiting times when dealing with dependencies of temporal nature.

To address the existing research gap, we first discussed four types of IUR and provided a way to model preference-based and context-based dependencies resulting from these IUR and context information. As considering IUR and context information could also lead to temporal-based dependencies, we further developed a concept for dealing with time especially waiting time by means of introducing waiting service classes and waiting services. Based on this, we presented a stateless as well as a stateful optimisation model to integrate these three types of dependencies. Additionally, by evaluating our approach, we were able to demonstrate its strengths and efficacy by means of a real-world scenario. In this regard, we could also show that in particular our stateless optimisation model could be solved in acceptable time for realistic problem sizes. We therefore contribute to the current body of knowledge in multi-user context-aware service selection.

Besides that, we also need to discuss some limitations of our work, which should be addressed in future research. First, we focused on service selection at planning time and, in this regard, we feel confident that modelling time as discrete seems sufficient in most cases. But there are certainly scenarios in which a consideration of time as quasi-continuous is required. This seems to be relevant, for instance, when selecting service objects at runtime of a process (e.g. re-planning during a city day trip). Although our approach could still consider such runtime scenarios by means of adjusting the factor c as needed (cf. Section 4.2), this would have a negative impact on the problem size and thus the computation time (cf. Scenario iv) of our performance evaluation in Section 6.2). Here, a promising idea may be the use of continuous instead of binary variables for time and waiting time in the stateless model. Second, although our performance evaluation – especially for our stateless model – mostly provided acceptable computation times from a planning point of view, we must account for the fact that the service selection problem is NP-hard which generally corresponds to an exponential development in computation time. Therefore, there are certainly situations where an approach providing an exact solution is not applicable. However, the aim of our work was not to present a computation time-optimised approach. Thus, further studies need to analyse whether and how time-optimised approaches resp. heuristic techniques (e.g. Alrifai et al., 2012; Canfora, Di Penta, Esposito, & Villani, 2008; Lewerenz, 2015) could be developed for our approach to consider IUR and context information in terms of multi-user processes. In addition, we focused on sequential processes. But existing works provide techniques to consider further control flow patterns like parallel, pick or conditional constructs (cf. Ardagna & Pernici, 2007; Yu et al., 2007), for instance, by using execution routes (cf. e.g. Alrifai et al., 2012; Ardagna & Pernici, 2007; Zeng et al., 2004). By this, our approach can easily be extended in future research to cope with such control flow patterns.

In conclusion, the provided multi-user service selection approach can serve as a promising first step for the aforementioned and further research in this interesting field.

## Notes

1. ‘An entity is a person, place, or object that is considered relevant to the interaction between a user and an application, including the user and the application themselves’ (Dey 2001, p. 5).

2.
https://www.tripadvisor.com/Tourism-g187309-Munich_Upper_Bavaria_Bavaria-Vacations.html, accessed July 2018.

4. The state transition function Φ for a state variable depends upon the type of the state variable and the corresponding context information.

5.
http://www.gurobi.com/, accessed July 2018.

6.
http://www.programmableweb.com/api/tripadvisor, accessed July 2018.

7.
http://www.programmableweb.com/api/google-places, accessed July 2018.

8. Favourite scores represent user favourites with respect to a certain category (e.g. type of restaurant) of an attribute.

## Appendix

### A. Notation

**Table:**

Notation	Description	Notation	Description
A	set of users participating in the process	$q_{e}^{\propto }$	quantified NFP value associated with dependency e and attribute ∝
$A_{e}^{\propto }$	set of users associated with dependency e and attribute ∝	$q_{a_{ij}}\left(ws_{a_{ik}}\right)$	quantified NFP value associated with world state $ws_{a_{ik}}$
$BS_{a_{i}}$	belief state of user a and service class i	$q_{e}^{\propto }\left(ws_{e}^{\propto }\right)$	quantified NFP value associated with world state $ws_{e}^{\propto }$ and attribute ∝
$bst_{a_{ik}}$	belief state tuple k of user a and service class i	$Q_{a}^{\propto }$	global end-to-end constraint of user a regarding attribute ∝
Dur	NFP duration	$s_{ij}$	service object j of service class i
$E_{a}^{\propto }$	set of dependencies of user a regarding attribute ∝	$s_{ij}^{*}$	waiting service
$F_{e}^{\propto }$	set of feasibility policies associated with dependency e and attribute ∝	$S_{i}$	service class/action i
I	number of service classes of the process	$S_{i}^{*}$	waiting service class
$IC_{a}^{Time}$	initial context of user a in terms of daytime at process start	$s_{e}^{\propto }$	binary decision variable associated with dependency e and attribute ∝
IUR	NFP Inter-User-Request	$U_{a_{ij}}$	utility value for user a and service object $s_{ij}$
$J_{i}$	number of service objects in service class i	${\hat{U}}_{e}^{\propto }$	utility value associated with time-independent dependency e and attribute ∝
M	the subset of NCA attributes, e.g., duration Dur, waiting time WT	${\bar{\overset{ˉ}{U}}}_{e}^{\propto }$	utility value associated with time-dependent dependency e and attribute ∝
N	the set of NCA and CA attributes (NFP)	$v\left(bst_{a_{ik}}\right)$	state variable for belief state tuple $bst_{a_{ik}}$
$N^{+}$	subset of attributes that need to be maximised	$w_{a}^{\propto }$	target weight of user a regarding attribute ∝
$N^{-}$	subset of attributes that need to be minimised	$ws_{a_{ik}}$	world state k of user a and service class i
O	the subset of CA attributes, e.g., IUR, *group discounts*	$ws_{e}^{\propto }$	world state for dependency e and attribute ∝
$P_{max}^{\propto }$	maximum quantified NFP value for attribute ∝ aggregated over all service classes	WT	NFP waiting time
$P_{i,\;max}^{\propto }$	maximum quantified NFP value for attribute ∝ of service class i	$x_{a_{ij}}$	binary decision variable for service object $s_{ij}$ and user a
$P_{min}^{\propto }$	minimum quantified NFP value for attribute ∝ aggregated over all service classes	$X_{e}^{\propto }$	set of service objects associated with dependency e and attribute ∝
$P_{i,\;min}^{\propto }$	minimum quantified NFP value for attribute ∝ of service class i	$y_{a_{ik}}$	binary decision variable for $ws_{a_{ik}}$
$q_{ij}^{\propto }$	quantified NFP value of service object $s_{ij}$ regarding attribute ∝	$y_{e}^{\propto }$	binary decision variable for $ws_{e}^{\propto }$
$q_{a_{ij}}^{\propto }$	quantified NFP value of user a for service object $s_{ij}$ regarding attribute ∝	$Z_{e}^{\propto }$	set of world states associated with dependency e and attribute ∝

### B. Stateless Optimisation Model

(1) $$\begin{array}{c} \underset{x_{a_{ij}};s_{e}^{\propto }}{\max}\underset{\alpha \in A}{\sum }\overset{I}{\underset{i=1}{\sum }}\underset{s_{ij}\in S_{i}}{\sum }U_{\alpha_{ij}}x_{\alpha_{ij}}+\underset{\alpha \in A}{\sum }\underset{\propto \in \hat{O}}{\sum }\underset{e\in E_{\alpha }^{\propto }}{\sum }{\hat{U}}_{e}^{\propto }\underset{x_{\alpha_{ij}}\in X_{e}^{\propto }}{\prod }x_{\alpha_{ij}}\\ +\underset{\alpha \in A}{\sum }\underset{\propto \in \bar{\overset{ˉ}{O}}}{\sum }\underset{e\in E_{\alpha }^{\propto }}{\sum }{\bar{\overset{ˉ}{U}}}_{e}^{a}s_{e}^{\propto }\underset{x_{\alpha_{ij}}\in X_{e}^{\propto }}{\prod }x_{\alpha_{ij}} \end{array}$$

(2) $$s.t.\;\underset{s_{ij}\in S_{i}}{\sum }x_{\alpha_{ij}}=1\;\forall i=1,\ldots ,I;\forall \alpha \in A$$

(3) $$\overset{I}{\underset{i=1}{\sum }}\underset{s_{ij}\in S_{i}}{\sum }q_{ij}^{\propto }x_{\alpha_{ij}}\leq Q_{\alpha }^{\propto }\;\;\forall \propto \in M;\forall \alpha \in A$$

(4) $$\underset{e\in E_{a}^{\propto }}{\sum }q_{e}^{\propto }\underset{x_{\alpha_{ij}}\in X_{e}^{\propto }}{\prod }x_{\alpha_{ij}}\leq \;Q_{\alpha }^{\propto }\;\;\forall \propto \in O;\forall a\in A$$

(5) $$\left[\begin{array}{c} \max_{\left\{\begin{array}{c} a\in A_{e}^{IUR}|\\ x_{a_{i^{\prime }j^{\prime }}}\in X_{e}^{IUR} \end{array}\right\}}\left(\overset{i^{\prime }-1}{\underset{i=1}{\sum }}\underset{s_{ij}\in S_{i}}{\sum }\underset{\propto \in \left\{Dur,WT\right\}}{\sum }q_{ij}^{\propto }x_{a_{ij}}+IC_{a}^{Time}\right)\\ -\min_{\left\{\begin{array}{c} a\in A_{e}^{IUR}|\\ x_{a_{i^{\prime }j^{\prime }}}\in X_{e}^{IUR} \end{array}\right\}}\left(\overset{i^{\prime }-1}{\underset{i=1}{\sum }}\underset{s_{ij}\in S_{i}}{\sum }\underset{\propto \in \left\{Dur,WT\right\}}{\sum }q_{ij}^{\propto }x_{a_{ij}}+IC_{a}^{Time}\right) \end{array}\right]*s_{e}^{IUR}=0$$

$$\forall e\in E_{a}^{IUR},\;{\bar{\overset{ˉ}{U}}}_{e}^{IUR}>0$$

(6) $$\left[\begin{array}{c} \max_{\left\{\begin{array}{c} a\in A_{e}^{IUR}|\\ x_{a_{i^{\prime }j^{\prime }}}\in X_{e}^{IUR} \end{array}\right\}}\left(\overset{i^{\prime }-1}{\underset{i=1}{\sum }}\underset{s_{ij}\in S_{i}}{\sum }\underset{\propto \in \left\{\begin{array}{c} Dur,\\ WT \end{array}\right\}}{\sum }q_{ij}^{\propto }x_{a_{ij}}+IC_{a}^{Time}\right)-\\ \min_{\left\{\begin{array}{c} a\in A_{e}^{IUR}|\\ x_{a_{i^{\prime }j^{\prime }}}\in X_{e}^{IUR} \end{array}\right\}}\left(\overset{i^{\prime }-1}{\underset{i=1}{\sum }}\underset{s_{ij}\in S_{i}}{\sum }\underset{\propto \in \left\{\begin{array}{c} Dur,\\ WT \end{array}\right\}}{\sum }q_{ij}^{\propto }x_{a_{ij}}+q_{i^{\prime }j^{\prime }}^{Dur}x_{a_{i^{\prime }j^{\prime }}}+IC_{a}^{Time}\right) \end{array}\right]*(1-s_{e}^{IUR})\geq 0$$

$$\forall e\in E_{a}^{IUR},\;{\bar{\overset{ˉ}{U}}}_{e}^{IUR}<0$$

(7) $$with\;x_{a_{ij}}\in \left\{0,\;1\right\};\;s_{e}^{\propto }\in \left\{0,1\right\}$$

### C. Stateful Optimisation Model

(1) $$\begin{array}{rl} & \underset{x_{a_{ij}};\;y_{a_{ik}};y_{e}^{\propto }}{\max}\underset{a\in A}{\sum }\overset{I}{\underset{i=1}{\sum }}\underset{s_{ij}\in S_{i}}{\sum }\underset{\begin{array}{c} ws_{a_{ik}}\\ \in BS_{a_{i}} \end{array}}{\sum }U\left(s_{ij},\;q_{a_{ij}}\left(ws_{a_{ik}}\right)\right)*x_{a_{ij}}*y_{a_{ik}}\\ & +\underset{a\in A}{\sum }\underset{\propto \in O}{\sum }\underset{\left\{\begin{array}{c} e\in E_{a}^{\propto }|\\ \left|A_{e}^{\propto }\right|>1\wedge \\ \left({\hat{U}}_{e}^{\propto }\neq 0\vee {\bar{\bar{U}}}_{e}^{\propto }\neq 0\right) \end{array}\right\}}{\sum }U\left(q_{e}^{\propto }\left(ws_{e}^{\propto }\right)\right)*y_{e}^{\propto } \end{array}$$

(2) $$s.t.\;\underset{s_{ij}\in S_{i}}{\sum }x_{a_{ij}}=1\;\forall i=1\;to\;I;\forall a\in A$$

(3) $$\underset{ws_{a_{ik}}\in BS_{a_{i}}}{\sum }y_{a_{ik}}=1\;\forall i=1\;to\;I;\forall a\in A$$

(4) $$\underset{ws_{a_{ik}}\in BS_{a_{i}}}{\sum }\left(y_{a_{ik}}*\underset{\left(s_{\left(i-1\right)j},\;ws_{a_{\left(i-1\right)k^{\prime }}}\right)\in \Theta_{ik}}{\sum }\left(x_{a_{\left(i-1\right)j}}*y_{a_{\left(i-1\right)k^{\prime }}}\right)\right)=1\;\forall a\in A;\;i\in \left[2;I\right]$$

(5) $$y_{e}^{\propto }-\underset{Z_{e_{k}}^{\propto }\in Z_{e}^{\propto }}{\sum }\underset{\left\{\begin{array}{c} a\in A_{e}^{\propto }|\\ x_{a_{ij}}\in X_{e}^{\propto } \end{array}\right\}}{\prod }x_{a_{ij}}\underset{ws_{a_{ik}}\in Z_{e_{k}}^{\propto }}{\sum }y_{a_{ik}}=0$$

$$\forall \propto \in O;\forall a\in A;\forall \begin{array}{c} e\in E_{a}^{\propto }\;with\;\left|A_{e}^{\propto }\right|>1\wedge \left({\hat{U}}_{e}^{\propto }\neq 0\vee {\bar{\overset{ˉ}{U}}}_{e}^{\propto }\neq 0\right) \end{array}$$

(6) $$\underset{Z_{e_{k}}\in Z_{e}}{\sum }\underset{\left\{\begin{array}{c} a\in A_{e}^{\propto }|\\ x_{a_{ij}}\in X_{e}^{\propto } \end{array}\right\}}{\prod }x_{a_{ij}}\underset{ws_{a_{ik}}\in Z_{e_{k}}}{\sum }y_{a_{ik}}=0$$

$$\forall \propto \in O;\forall a\in A;\forall \begin{array}{c} e\in E_{a}^{\propto }\;with\;\left|A_{e}^{\propto }\right|>1\wedge F_{e}^{\propto }\neq \emptyset \end{array}$$

(7) $$\overset{I}{\underset{i=1}{\sum }}\underset{s_{ij}\in S_{i}}{\sum }q_{a_{ij}}^{\propto }*x_{a_{ij}}\leq Q_{a}^{\propto }\;\;\forall \propto \in M\;and\;\forall a\in A$$

(8) $$\overset{I}{\underset{i=1}{\sum }}\underset{s_{ij}\in S_{i}}{\sum }\underset{ws_{a_{ik}}\in BS_{a_{i}}}{\sum }q_{a_{ij}}^{\propto }\left(ws_{a_{ik}}\right)*x_{a_{ij}}*y_{a_{ik}}+\underset{\left\{\begin{array}{c} e\in E_{a}^{\propto }|\\ \left|A_{e}^{\propto }\right|>1\wedge \\ \left({\hat{U}}_{e}^{\propto }\neq 0\vee {\bar{\overset{ˉ}{U}}}_{e}^{\propto }\neq 0\right) \end{array}\right\}}{\sum }q_{e}^{\propto }\left(ws_{e}^{\propto }\right)*y_{e}^{\propto }\leq Q_{a}^{\propto }$$

∀∝∈O;∀a∈A

(9) $$with\;x_{a_{ij}}\in \left\{0,\;1\right\};\;y_{a_{ik}}\in \left\{0,\;1\right\};y_{e}^{\propto }\in \left\{0,\;1\right\}$$

### D. Performance Evaluation: Basic Configuration

**Table:**

Parameter	Regular Service Class	Waiting Service Class
No. of service classes	4	4
No. of service objects	6	5
NCA duration	randomly selected in the interval [15;60] min in steps of 15 min	
NCA waiting time		0–60 min in steps of 15 min
NCA price	randomly selected within Gaussian distribution X ~ N(10, 25) €	
CA distance	randomly selected in the interval [47.95;48.35] for latitude and [11.25;11.90] for longitude	
No. of users	3	
No. of IUR	4 IUR per user (one of each type), with randomly generated utility in the interval [0;0.3] for complementary IUR and [−0.3;0] for conflicting IUR	
Users’ target weights regarding NCA and CA attributes	same target weight for duration, waiting time, price, distance and IUR for each user	
Users’ constraints regarding NCA and CA attributes	max. possible aggregated NFP value for duration, waiting time, price and distance for each user	
Users’ initial context (i.e. GPS position)	randomly selected in the interval [48.06;48.25] for latitude and [11.36;11.72] for longitude for each user	
